# Supplementary material for: Donor and host photoreceptors engage in material transfer following transplantation of post-mitotic photoreceptor precursors
Source: Nat Commun. 2016 Oct 4;7:13029. doi: 10.1038/ncomms13029 (PMC5059468; doi:10.1038/ncomms13029)
Supplement: Supplementary Information — Supplementary Figures 1-2 [file ncomms13029-s1.pdf]

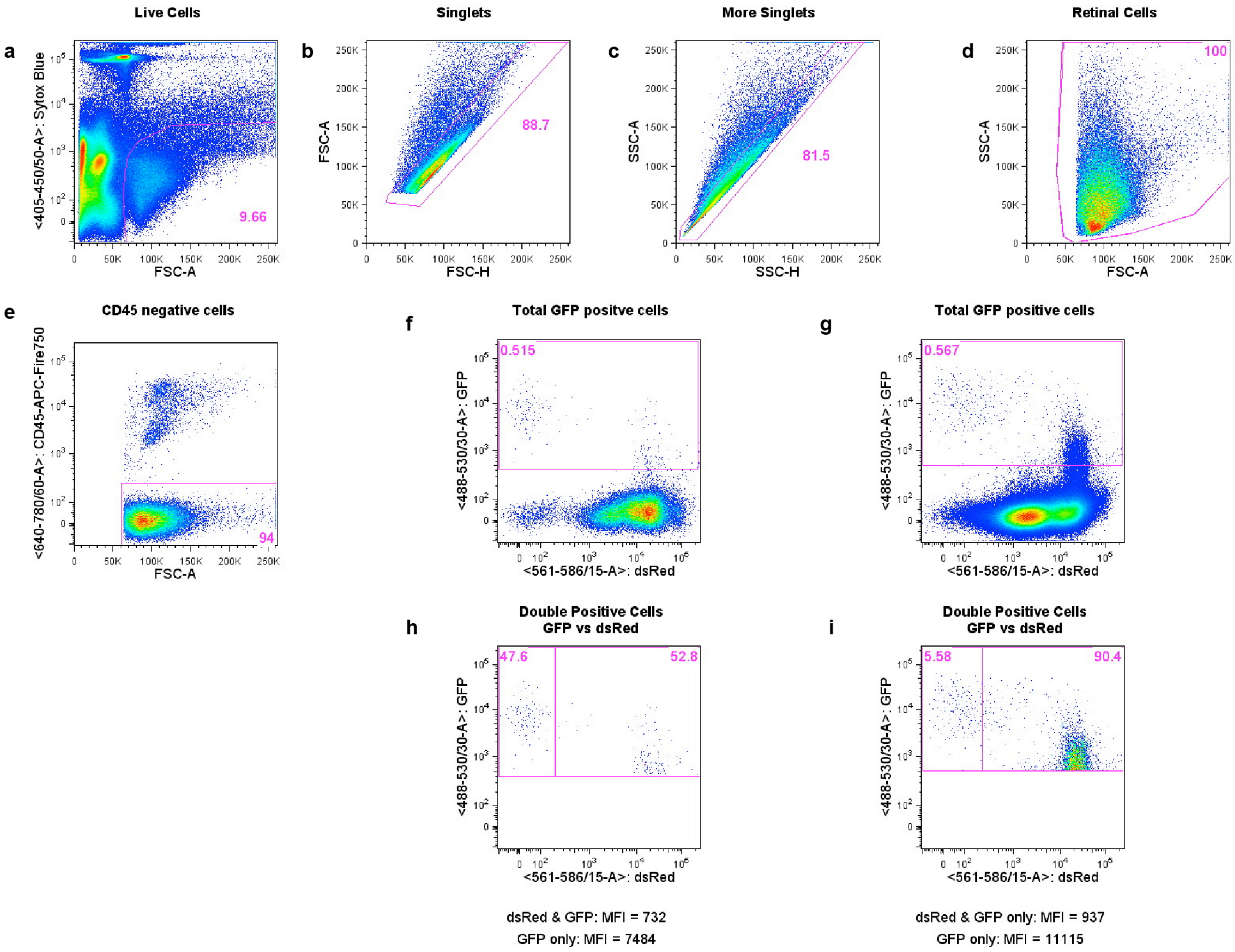

Supplementary Figure 1. Flow cytometry gating strategy for analysis of host (*DsRed*) and donor-derived (*NrlGFP*) reporter proteins following transplantation. This example displays a *DsRed* host retina examined by flow cytometry 5-6 wks post-transplantation of *NrlGFP* post-mitotic photoreceptor precursor donor cells. **a,** a representative plot showing a dissociated retina stained for Sytox Blue dead cell stain used to exclude dead cells and cellular debris and identify the live cell population. **b,c,** plots showing the exclusion of cellular aggregates to isolate single cells. **d,** a representative plot depicting the relative size and granularity of single, live retinal cells. **e,** CD45, leukocyte common antigen (LCA), staining was subsequently used to gate on the CD45$^-$ cell population. **f, g,** examples of two sample plots showing total GFP$^+$ cells (*pink box*). The gate was set based on controls presented in figure 3 in the main text. **h, i,** subsequent gating of the two examples showing the % of GFP$^+$-only (*left pink box*) and GFP$^+$/DsRed$^+$ (*right pink box*). In the first example, a similar number (~50%) of GFP$^+$-only and GFP$^+$/DsRed$^+$ were observed (**h**). The other example displays a much greater number (~90%) of double positive GFP$^+$/DsRed$^+$ when compared to GFP$^+$-only cells (**i**). Abbreviation: MFI (mean fluorescent intensity).

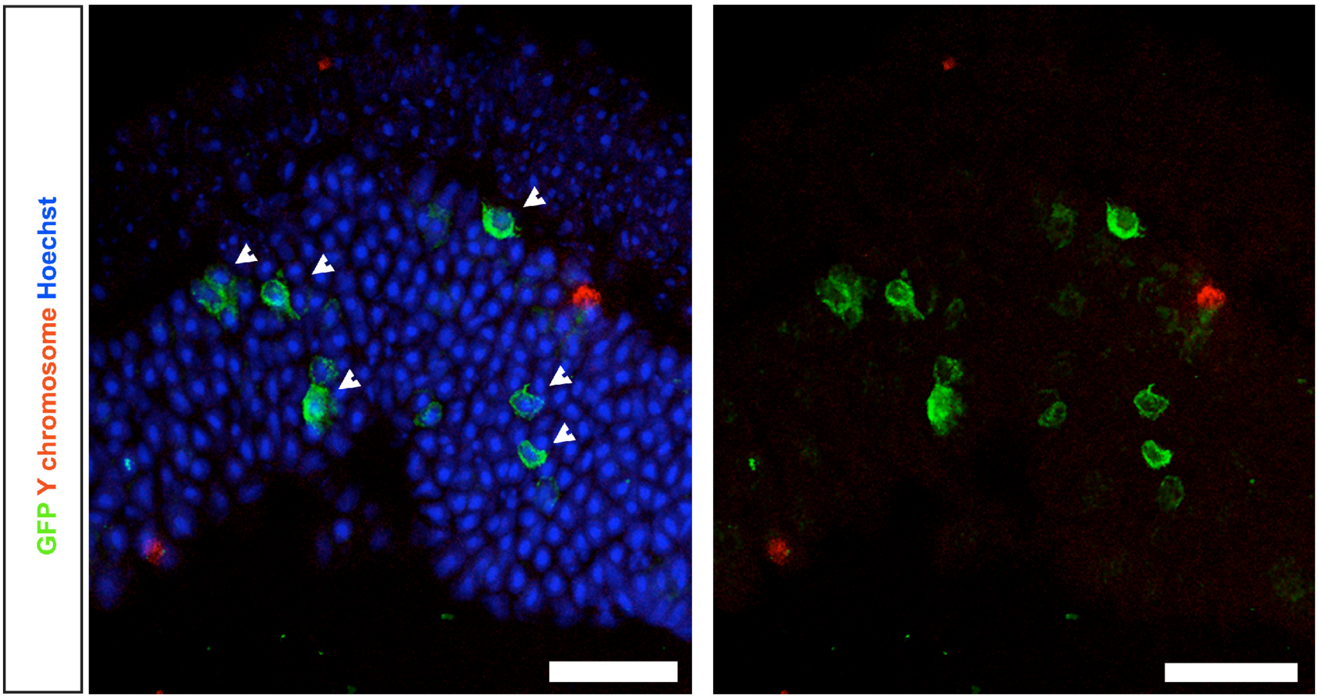

Supplementary Figure 2. Fluorescent In Situ Hybridisation (FISH) for the male Y-chromosome following transplantation of male NrlGFP donor cells into adult female wildtype hosts. Confocal projection images show example of several GFP^{+} (*green*) cells within female host that lacked Y-chromosome staining (*red*). Images show individual and/or combined channels for the same region of interest. Scale bar 25μm.
